# Development and Evaluation of a SYBR Green-Based Real Time RT-PCR Assay for Detection of the Emerging Avian Influenza A (H7N9) Virus

**DOI:** 10.1371/journal.pone.0080028

**Published:** 2013-11-20

**Authors:** Zheng Zhu, Huan Fan, Xian Qi, Yuhua Qi, Zhiyang Shi, Hua Wang, Lunbiao Cui, Minghao Zhou

**Affiliations:** 1 Institute of Pathogenic Microbiology, Jiangsu Provincial Center for Disease Control and Prevention, Key Laboratory of Enteric Pathogenic Microbiology, Ministry of Health, Nanjing, China; 2 Department of Acute Infectious Diseases Control and Prevention, Jiangsu Provincial Center for Disease Control and Prevention, Nanjing, China; University of Texas Medical Branch, United States of America

## Abstract

Most recently a novel avian-origin influenza A (H7N9) virus emerged in China and has been associated with lots of human infection and fatal cases. Genetic analysis of the viral genome revealed that this reassortant virus might be better adapted to humans than other avian influenza viruses. Molecular diagnostic methods are thus urgently needed in public health laboratories. In this study, a SYBR green-based one-step real time reverse transcription-PCR (RT-PCR) was developed to detect the novel H7N9 virus. The primer pairs on the basis of the hemagglutinin and neuraminidase gene sequences of H7N9 viruses amplified subtype-specific fragments with Tm values of 80.77±0.06°C for H7 and 81.20±0.17°C for N9 respectively. The standard curves showed a dynamic linear range across 6 log units of RNA copy number (10^6^ to 10^1^ copies/ µl) with a detection limit of 10 copies per reaction for both H7 and N9 assays by using serial ten-fold diluted in-vitro transcribed viral RNA. In addition, no cross-reactivity was observed with seasonal H1N1, H1N1 pdm09, H3N2, H5N1 and H9N2 viruses as well as other human respiratory viruses. When the assay was further evaluated in H7N9 virus infected clinical samples, positive amplification signals were obtained in all of the specimens with the accordance between H7 and N9 assays. Therefore, the established SYBR green-based real time RT-PCR assay could provide a rapid, sensitive, specific and reliable alternative approach with lower costs for high throughput screening of suspected samples from humans, animals and environments in first line public health laboratories.

## Introduction

On 31 March 2013, three died urban residents from eastern China were reported to be infected with a novel avian-origin influenza A virus [1]. Sequencing analyses revealed that hemagglutinin (HA) and neuraminidase (NA) genes of this emerging reassortant virus belonged to H7 and N9 subtypes of influenza A virus respectively, while six internal genes were closely related to those of avian H9N2 viruses [1]. An increasing number of patients infected with H7N9 virus subsequently occurred in many provinces of China. As of 30 May 2013, a total of 132 laboratory-confirmed cases of human infection with avian influenza A (H7N9) virus have been reported, 37 of whom have died [2]. The novel virus infection is associated with severe clinical symptoms characterized by rapidly progressive pneumonia, acute respiratory distress syndrome (ARDS) and multiorgan failure [1], [3], [4]. Since H7N9 virus has not been detected previously in human beings or animals, the possible source of infections and the reservoirs of this virus remain unclear. Moreover, sporadic human infections with H7N9 virus to date raise more important questions that whether this virus will persistently evolve for better adaption to humans, and whether limit or even sustained human-to-human transmissions will occur. Such questions induce global public health concerns.

For more effective prevention and control of H7N9 virus infection in humans, development of a rapid, sensitive and specific molecular diagnostic assay is urgently needed in many laboratories. Conventional virus isolation from cell lines is time-consuming and thus delays the detection efficiency. Because specific antibodies in response to virus infection are generally produced at about 3∼4 weeks in the bodies of infected patients, monoclonal antibody-based ELISA is not well suitable as an early diagnostic tool in the initial stage of the disease. In contrast, viral DNA or RNA from different clinical samples is an appropriate detection target during the acute phase of an infection or even before the onset of illness. Various detection methods targeting viral nucleic acids have been developed for the rapid identification of viral diseases, including conventional RT-PCR [5], real time RT-PCR [6], microarray examination [7], electrospray ionization mass spectrometry [8] and loop-mediated isothermal amplification (LAMP) [9], of which real-time RT-PCR has become the most popular molecular diagnostic approach in clinical laboratories owing to its simplicity, high sensitivity and specificity. Real-time RT-PCR assays are usually carried out with the use of hydrolysis probe (Taqman) or SYBR green fluorescence dye. Compared to the Taqman probe, SYBR green-based real time RT-PCR assay can achieve the same detection efficiency with lower costs and thus has been also widely applied in the detection of a variety of viruses [10], [11], [12], [13], [14].

In present study, we developed a rapid, sensitive and specific SYBR green-based one-step real time RT-PCR assay for the molecular diagnosis of H7N9 virus. Compared with cell culture strategy and immunological assays, this nucleic acid-targeting diagnostic procedure has great advantages in virus detection during the early stage of infection, which could facilitate enhanced epidemiological surveillance of suspected human infection with the emerging influenza virus as well as dynamic monitoring of viral gene sequence evolution. Since the original natural reservoirs of H7N9 virus have not been definitely determined, the established detection assay could also provide a simple and economic approach for H7N9 virus screening in large scale specimens from various animals, including chickens, ducks, geese, pigeons, quails and wild birds.

## Materials and Methods

### Ethics Statement

This study was approved by the Ethics Committee of Jiangsu Provincial Center for Disease Control and Prevention. Written informed consent for the use of the clinical samples was obtained from all of the patients involved in this study.

### Clinical Samples and Virus Isolation

The respiratory specimens obtained from patients with influenza-like illnesses were maintained in the viral transportation medium (Yocon Biotech, Beijing, China) and then detected for H7N9 virus infection according to the protocol provided by World Health Organization (WHO) [15]. A total of 7 clinical samples (5 throat-swabs, 1 sputum and 1 tracheal aspirate) from H7N9-infected patients were collected and stored at −70°C until further analysis. Virus isolation was performed in Madin-Darby canine kidney cells for 7 to 14 days. The positive cell culture supernatant was harvested and stored at −70°C. A H7N9 virus isolate (A/Nanjing/1/2013) was used as the reference virus.

### RNA Extraction

Viral RNA was extracted from 200 µl of cell culture supernatants or respiratory specimens using QIAsymphony Virus/Bacteria Mini Kit combined with the QIAsymphony SP instrument (Qiagen, Germany), following the manufacturer’s instructions. The extracted RNA was eluted in 60 µl of nuclease-free water and stored at −70°C until use.

### Sequence Analysis and Primer Design

The primers of HA and NA genes were designed based on the first three genomic sequences of the emerging H7N9 virus (A/Anhui/1/2013, A/Shanghai/1/2013 and A/Shanghai/2/2013) deposited in the GISAID EpiFlu database, using the software Primer3 version 4.0. To increase the wide spectrum compatibility of the primers for other strains of H7 or N9 subtype influenza A virus, the mixed base was introduced into each primer. In detail, all of the H7 or N9 gene sequences of the known circulating virus strains from Asian origin were downloaded from the NCBI’s influenza virus resource database [16] and subsequently aligned with the primers by BLAST. If a base close to the 3′ end of each primer was different from that of other H7 or N9 subtype viral gene sequences, the original base would be substituted by a mixed base to ensure full complementation of several bases close to the 3′ end of each primer with the templates as possible. Additionally, the number of mixed base sites within each primer was not allowed more than three positions as a general rule.

### SYBR Green-based Real Time RT-PCR Assay

The one-step real time quantitative RT-PCR assays were performed to amplify the HA and NA genes of H7N9 virus respectively using SuperScript III Platinum SYBR Green One-Step qRT-PCR kit (Invitrogen, CA, USA). The assay was carried out in a 10 µl reaction mixture containing 5 µl of 2× SYBR Green Reaction Mix, 0.8 µM of each primer, 0.2 µl of ROX Reference Dye, 0.2 µl of SuperScript III RT/Platinum *Taq* Mix and 1 µl of purified RNA. The optimized thermal cycling conditions were as follows: a reverse transcription step at 50°C for 10 min, an initial denaturation step at 95°C for 5 min, 40 cycles of PCR amplification at 95°C for 15 sec, 60°C for 20 sec, and 72°C for 30 sec, followed by a melting curve analysis program according to the instrument documentation. All real time RT-PCR reactions were conducted in triplicate on the ABI Prism 7900HT Sequence Detection System (Applied Biosystems, CA, USA). The RT-PCR amplicons were subjected to capillary electrophoresis analysis on the QIAxcel System (Qiagen, Germany) to confirm their specificity.

### In-vitro Transcription of Viral RNA

The viral HA and NA genes were amplified from a H7N9 virus isolate A/Nanjing/1/2013 using the following primers containing T7 promoter sequence in the reverse sites: HA-For: 5′-CCTGGTATTCGCTCTGATTGC-3′, HA-Rev: 5′- ACTCGTTAATACGACTCACTATAGGGAGGCACCGCATGTTTCCATTCT-3′; NA-For: 5′-TCTATGCACTTCAGCCACTG-3′, NA-Rev: 5′- ACTCGTTAATACGACTCACTATAGGGAGCCATCAGGCCAGTTCCATTG-3′. The amplified HA and NA gene segments were then in-vitro transcribed with T7 RNA polymerase (TaKaRa, Dalian, China) according to the manufacturer’s instructions. Finally, the synthesized RNA transcripts were purified, quantified and mixed with equal-molar amounts.

### Detection Limit and Standard Curve

The detection limit of the SYBR green real time RT-PCR assay was determined by testing serial ten-fold dilutions of the in-vitro transcribed viral RNA ranging from 10^6^ to 10^0^ copies/µl. Then the obtained cycle-threshold (Ct) values were plotted against the amount of RNA copy number to construct standard curve.

### Specificity of the Assay

The specificity of the assay developed to detect H7N9 virus was evaluated on viral RNA from known genetically and clinically related influenza A viruses including seasonal H1N1 (A/Nanjing/4/2009), H1N1 pdm09 (A/Jiangsu/S61/2009), H3N2 (A/Nanjing/1/2009), H5N1 (A/Jiangsu/1/2007) and H9N2 (A/swine/Jiangxi/wx2/2004), as well as other human respiratory viruses including influenza B virus, parainfluenza viruses (types 1, 2, 3 and 4), coronaviruses (HKU1, OC43, 229E and NL63), rhinovirus (China/JS/YC29/2011), respiratory syncytial viruses (types A and B) and enterovirus 71 (Nanjing08-1).

## Results

### Sequence Analysis and Primer Design

**Figure 1:**
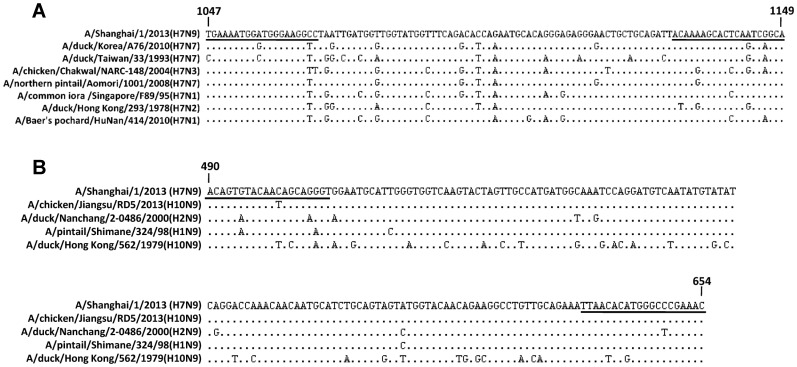
Sequence analysis and primer design. The regions targeted by primer pairs are nucleotide 1047 to 1149 for HA sequence (A) and nucleotide 490 to 654 for NA sequence (B) of H7N9 virus. The underlined sequences were used for the primer design. Sequence alignments were conducted between the primers and 135 H7 and 36 N9 subtype full-length viral gene sequences from NCBI’s influenza virus resource database, some representatives of which are shown here. Nucleotides identical to those of A/Shanghai/1/2013 (H7N9) strain are indicated as dots. A mixed base was used close to the 3′ end of each primer when a different nucleotide was encountered. The modified primer sequences were listed in Table 1.

We designed specific forward and reverse primers for the detection of HA and NA genes of H7N9 virus based on the viral genomic sequences from the first three infected patients published in the GISAID EpiFlu database. The regions targeted by primer pairs were nucleotide 1047 to 1149 for HA sequence and nucleotide 490 to 654 for NA sequence respectively, as shown in Figure 1. In order to increase the detection scope of the primers for known H7 or N9 subtype avian influenza viruses, 135 H7 and 36 N9 subtype full-length viral gene sequences from Asian lineage were downloaded from NCBI’s influenza virus resource database and then aligned with the primers by BLAST. A mixed base was used close to the 3′ end of each primer when a different nucleotide was found between the primers and the downloaded viral gene sequences (Figure 1). Detailed primer sequences were listed in Table 1.

**Table 1:** The primer sequences used for detection of influenza A H7N9 virus.

Primer^a^	Sequence (5′-3′)^b^
H7F	TGAAAATGGVTGGGAAGGYY
H7R	TGCCGATTGRGTGCTYTTRT
N9F	ACAGTGTACAAYAGCARRGT
N9R	GTTTCGRGCCCAYGTRTTAA

a F: Forward; R: Reverse.

b V: A/C/G; Y: C/T; R: A/G.

### Validation of SYBR Green-based Real Time RT-PCR Assays

**Figure 2:**
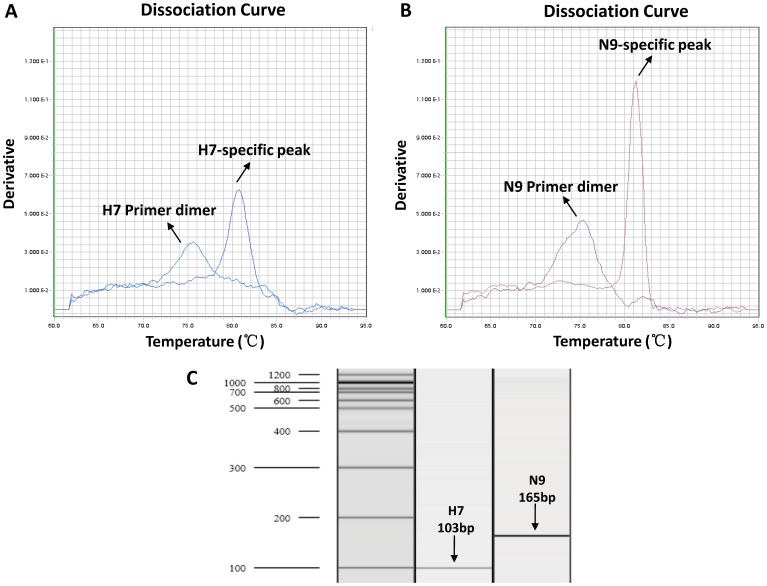
Melting curve analysis and confirmation of H7- and N9-specific amplicons from SYBR green-based real time RT-PCR assays. The specific peaks of H7 (A) and N9 (B) amplicons as well as primer dimers are shown. Capillary electrophoresis analysis was then carried out to confirm the lengths of specific amplicons (C).

We employed the genomic RNA as the template purified from a H7N9 virus isolate (A/Nanjing/1/2013) for the initial validation experiments. The optimal annealing temperature was set at 60°C, while optimal primer concentrations were 0.8 µM in a 10 µl of reaction mixture. After 40 amplification cycles, the melting curve analysis revealed that the mean melting temperatures (Tm) of the specific amplicons were 80.77±0.06°C for H7 and 81.20±0.17°C for N9, quite different from those of primer-dimers (Figure 2A and 2B). The RT-PCR amplification segments were then confirmed with a length of 103 bp for H7 and 165bp for N9 by capillary electrophoresis analysis (Figure 2C).

### Detection Limit and Standard Curve

**Figure 3:**
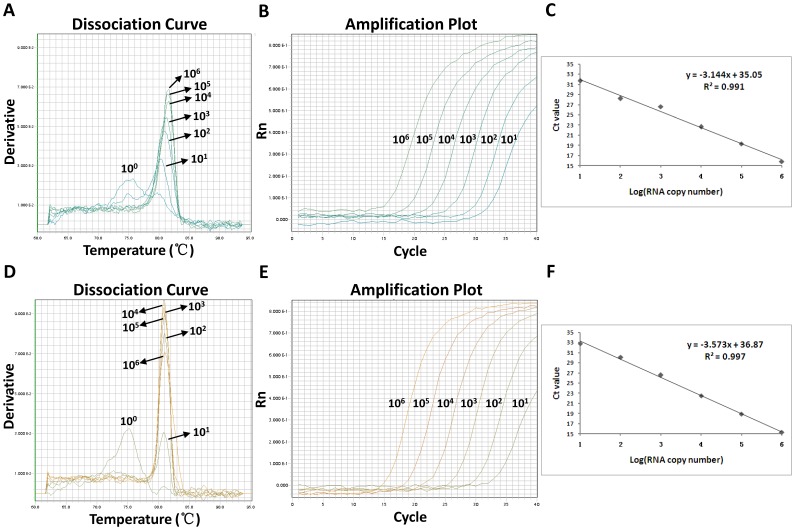
Detection limit and standard curve. The detection limit was determined based on serial ten-fold dilutions of in-vitro transcribed viral RNA ranging from 10^6^ to 10^0^ copies/µl. Melting curves and amplification plots are indicated for H7 (A, B) and N9 (D, E) assays. The standard curves of H7 (C) and N9 (F) assays were constructed upon the Ct values against the amount of RNA copy number.

Using serial ten-fold diluted in-vitro transcribed viral RNA, we observed that real time RT-PCR assays with 10^6^ to 10^1^ RNA copies as the reaction templates could generate positive amplification signals for both H7 and N9 examinations, but not with 10^0^ RNA copy (Figure 3A and 3D). The Ct values corresponding to 10^1^ RNA copies per reaction were 31.70±0.14 (H7) and 32.80±0.76 (N9) respectively (Figure 3B and 3E). The standard curves showed a dynamic linear range across at least 6 log units of RNA copy number. Linear regression analysis revealed that the correlation coefficients (R^2^) were 0.991 with a slope value of 3.144 for H7 assay and 0.997 with a slope value of 3.573 for N9 assay (Figure 3C and 3F).

### Specificity of the Assay

The specificity of SYBR green real time RT-PCR assay was tested on genomic RNA from a panel of viral positive isolates. Negative amplification signals were obtained in both assays for several known subtypes of human influenza A virus, including seasonal H1N1, H1N1 pdm09, H3N2, H5N1 and H9N2 viruses (Table 2). And there were also no positive amplification signals produced when the H7 and N9 assays were applied in the detection of other human respiratory viruses (Table 2).

**Table 2:** Known human respiratory viruses used for testing the specificity of the SYBR green real time RT-PCR assay.

Virus	SYBR green RT-PCR result	
	H7 assay	N9 assay
Influenza A virus		
Seasonal H1N1	Negative	Negative
H1N1 pdm09	Negative	Negative
H3N2	Negative	Negative
H5N1	Negative	Negative
H9N2	Negative	Negative
Influenza B virus	Negative	Negative
Parainfluenza virus		
Type 1	Negative	Negative
Type 2	Negative	Negative
Type 3	Negative	Negative
Type 4	Negative	Negative
Coronavirus		
hCoV-HKU1	Negative	Negative
hCoV-OC43	Negative	Negative
hCoV-229E	Negative	Negative
hCoV-NL63	Negative	Negative
Rhinovirus	Negative	Negative
Respiratory syncytial virus		
Type A	Negative	Negative
Type B	Negative	Negative
Enterovirus 71	Negative	Negative

### Evaluation of the SYBR Green Real Time RT-PCR Assays in Clinical Samples

**Figure 4:**
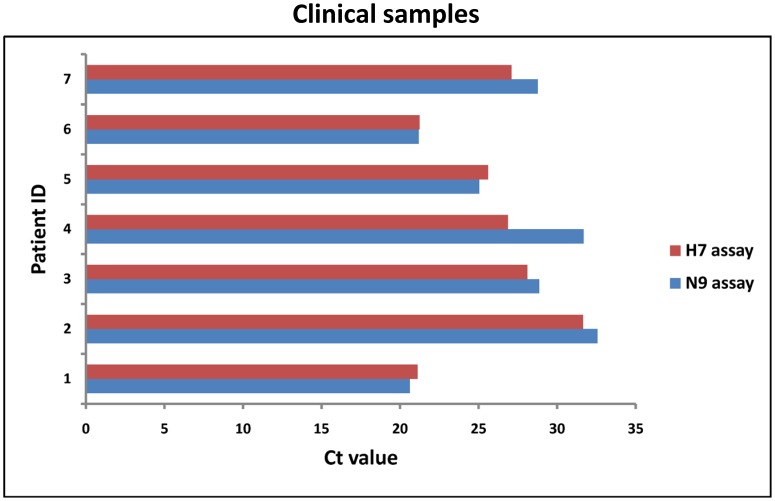
Real time RT-PCR results using respiratory specimens from seven laboratory-confirmed patients with H7N9 virus infection. The Ct values obtained from H7 and N9 assays for every clinical sample are presented.

To evaluate the ability of the developed assays to detect the clinical samples, the respiratory specimens from seven previously confirmed patients infected with H7N9 virus were used for analysis. Real time RT-PCR results showed that positive signals were produced in all of the samples for both H7 and N9 examinations. Moreover, the Ct value obtained from H7 assay was well comparable with that obtained from N9 assay for each individual infected patient (Figure 4).

## Discussion

Influenza A virus, a negative-sense RNA virus, belongs to the family *Orthomyxoviridae*. It is subtyped on the basis of HA and NA glycoproteins on the surface of the virus particle, which are antigenically classified into 16 (H1 to H16) and 9 (N1 to N9) subtypes, respectively. Human infections with influenza A virus are generally confined to 3 HA subtypes (H1, H2 and H3) and 2 NA subtypes (N1 and N2), whereas domestic poultry and wild birds usually serve as the natural reservoirs for the other subtypes [17]. Cross-species transmissions of avian influenza viruses from poultry to humans have been reported before and only focused on H5N1 [18], H7N2 [19], H7N3 [20] and H7N7 [21] subtypes, among which highly pathogenic H5N1 virus has caused 634 infections and 371 deaths as of 12 March 2013 [22], and H7N7 virus infection was associated with a fatal case in Netherland [21].

Most recently, a newly identified avian-origin reassortant influenza A (H7N9) virus emerged in China and has led to an increasing number of human infection and fatal cases. Although at this moment there is no potent evidence of sustained human-to-human transmission, genetic analysis suggests that several amino acid changes in viral HA and PB2 RNA polymerase genes improve the ability to bind human-type receptors and facilitate the efficient replication in mammals [22], highlighting the great threat to human beings. Furthermore, the real sources of infection and the reservoirs of this novel virus are still uncertain despite the fact that one virus isolate has been acquired from a chicken [3]. Many other questions, such as modes of transmission, viral incubation period, the optimal clinical specimens for laboratory confirmation and the spectrum of clinical illness, remain to be further determined [23]. Therefore, a lot of measures must be taken to effectively prevent and control the outbreak of H7N9 virus infection, of which development of a rapid molecular diagnostic assay is the top priority in public health laboratories.

Here we developed a SYBR green-based real time RT-PCR assay for rapid identification of this novel H7N9 virus. Although the primers were designed based on the sequences from the initial three isolates, the H7 and N9 primer pairs used in this study are suitable for the detection of all of the new reassortant H7N9 virus stains available in the GISAID EpiFlu database by sequence alignment. Moreover, the introduction of mixed bases into primers could make it possible to detect other known circulating H7Nx and HxN9 influenza viruses. Since SYBR green dye can intercalate into non-specific DNA such as primer dimers [10], the results are usually analyzed in terms of amplification plots combined with melting curves to discriminate non-specific amplification. H7- and N9-specific amplicons in this assay had unique Tm values, significantly different from those of primer dimers to avoid false positive results. In-vitro transcribed viral RNA is very useful for eliminating the need for handling contagious virus isolates and has been preferentially applied in testing sensitivity of various virus diagnostic assays [11], [12], [14]. With the use of in-vitro RNA transcripts of HA and NA genes of H7N9 virus, the assay was found to detect H7 and N9 genes with a detection limit of 10 copies per reaction, similar to the Taqman probe-based assay presented by Corman et al [24]. The specificity of the assay was subsequently established through testing a panel of previously known influenza A virus subtypes as well as other human respiratory viruses. No specific amplification signals were observed in all of the reactions with the viruses above as the templates, indicating the high specificity of the assay. To evaluate the practical use of the SYBR green-based real time RT-PCR assay, we collected clinical samples from seven H7N9 infected patients during the acute stage of the disease. All of the samples were readily amplified in H7 and N9 examinations by this method, suggesting its effectiveness in clinical practice. Besides the diagnosis of clinically suspected samples, this developed real time RT-PCR assay has the potential in dynamic monitoring of viral replication based on series specimens from individual patient with H7N9 virus infection, thus guiding the therapeutic strategy in clinic. Large scale specimens need to be collected to further evaluate the diagnostic robustness of the assay.

Considering the high variation frequency in sequences of influenza viruses, we didn’t develop a Taqman probe-based assay for the detection of the emerging H7N9 virus. Reduced sensitivity and elevated false negative results have been often encountered in probe-based assays designed to detect highly variable viral genomes due to the fact that the presence of mutations within the probe target region can prevent annealing of the probe and subsequent hydrolysis [25], [26], [27], [28]. However, SYBR green-based RT-PCR assays are less influenced by mismatches because of probe independency. We also took other advantages of SYBR green quantification into account including the comparable amplification performance with Taqman probes but the relative simplicity and lower running costs, which might be more appropriate for use in first line clinical laboratories.

In summary, the present SYBR green-based real time RT-PCR assay proved to be a powerful tool for rapid, sensitive and specific detection of the emerging H7N9 virus. It could provide a simple and economic alternative approach for high throughput screening of suspected samples from humans, animals and environments, thus allowing better pandemic preparedness.
